# A cyclic dipeptide from the Chilean hazelnut cotyledons (*Gevuina avellana* Mol., Proteaceae)

**DOI:** 10.1038/s41598-020-63983-9

**Published:** 2020-04-27

**Authors:** Guillermo Schmeda-Hirschmann, Jean Paulo de Andrade, Felipe Jiménez-Aspee, Daniel Mieres-Castro

**Affiliations:** 1 Laboratorio de Química de Productos Naturales, Instituto de Química de Recursos Naturales, Universidad de Talca, Campus Lircay, Talca, Chile; 2 Núcleo Científico Multidisciplinario, Dirección de Investigación, Universidad de Talca, Campus Lircay, Talca, Chile; 3 Departamento de Ciencias Básicas Biomédicas, Facultad de Ciencias de la Salud, Universidad de Talca, Campus Lircay, Talca, Chile; 4 Present Address: Institute of Nutritional Sciences, Department of Food Biofunctionality, University of Hohenheim, Garbenstrasse 28, 70599 Stuttgart, Germany

**Keywords:** Biological sciences, Plant sciences, Secondary metabolism

## Abstract

The Chilean hazelnut (*Gevuina avellana* Mol., Proteaceae) is a southern South American nut consumed as a snack and included in different preparations of traditional Chilean cuisine. Recently we described the fatty acid profile, oxylipins, phenolic compounds, as well as the antioxidant capacity. The main compounds of the phenolic-enriched extract were only tentatively identified by spectrometric means. In the present work, we describe the isolation and full characterization of a cyclic dipeptide cyclo(Arg-Trp) and other compounds from the phenolic enriched extracts of the *G. avellana* cotyledons. Compounds were isolated by means of counter-current chromatography and structures were established by spectroscopic and spectrometric methods. This is the first report on small peptides in *G. avellana* and adds evidence on the possible beneficial effects of this nut in human health.

## Introduction

**Figure 1:**
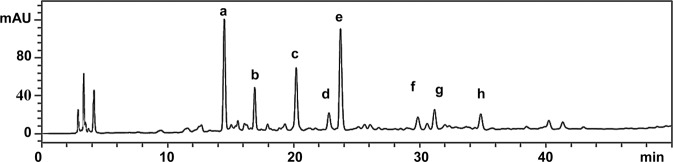
HPLC-DAD chromatogram of the PEE from  Chilean hazelnuts. Compounds. (**a**) caffeic acid hexoside; (**b)** hydroxymethylfurfural; (**c**) main compound: tryptophan; (**d**) phenyl caffeate; (**e**) cyclo (Arg-Trp); (**f**) hydroxybenzoic acid; (**g**) sinapic acid hexoside; (**h**) benzoic acid.

Gathering fruits from wild Chilean hazelnuts (*Gevuina avellana* Mol., Proteaceae) is a relevant economic activity in the rural areas of central and southern Chile^1,2^. The roasted cotyledons are consumed as snacks, milled to make flour for confectionary and pastry, or to prepare hot drinks as coffee substitutes. The fatty acid, oxylipin and phenolic compounds in this species have been recently published, and the phenolic-enriched extracts (PEE) of the cotyledons were assessed on enzymes associated with metabolic syndrome^2^. The HPLC-DAD analysis of the PEE showed several compounds, identified as caffeic acid hexoside (**a**), hydroxymethylfurfural (**b**), phenyl caffeate (**d**), hydroxybenzoic acid (**f**) and sinapic acid hexoside (**g**), as well as two UV absorbing peaks (**c** and **e**) with UV maxima at 280 and 222 nm (Fig. 1). When the PEE was analyzed by HPLC-MS/MS using the negative ionization mode, no ions were detected for peaks **c** and **e**, suggesting the occurrence of nitrogen-containing compounds. Little is known on the occurrence of nitrogen-containing compounds in Proteaceae. The alkaloids bellendine, isobellendine and darlingine were the most common compounds in *Bellandena montana*, *Darlingia darlingiana* and *Knightia* species^3^. Additional alkaloids were reported from *Triunia erythrocarpa*^4^. No information is available on nitrogen-containing compounds from *Gevuina avellana* other than the amino acids from defatted nut after hydrolysis^5^. Hence, the aim of the present study was to isolate and characterize the main nitrogen-containing compound from the PEE of *G. avellana* cotyledons.

## Materials and Methods

### Chemicals

Acetonitrile (ACN), methanol (MeOH), chloroform, 1-butanol (1-BuOH), ethyl acetate (EtOAc), petroleum ether (PE), *tert*-buthyl methyl ether (TBME) and formic acid were purchased from Merck (Darmstadt, Germany). Amberlite XAD-7 HP resin, ammonium hydroxide, sulfuric acid, formic acid, and trifluoroacetic acid, were obtained from Sigma-Aldrich (St. Louis, MO, USA). Ultrapure water was obtained from a Barnstead EasyPure water system (Thermo Scientific, Marietta, OH, USA).

### Plant material

The roasted cotyledons from *Gevuina avellana* were purchased at Contulmo, Región del Bio-Bio, Chile, in 2018 and 2019. The fruits were identified by Dr. Patricio Peñailillo, Botanist from the Instituto de Ciencias Biológicas, University of Talca. Vouchers of the plant and fruits are deposited at the institutional herbarium under the numbers GSH 40–1 for the 2018 sample and GSH 40–2 for the 2019 collection, respectively. The phenolic-enriched extract (PEE) was prepared as described in Pino-Ramos *et al*.^2^. In addition, a sample of air-dried raw cotyledons (914 g) was powdered and extracted two times with aqueous sulfuric acid (pH 2–3) (4 L) under sonication (20 min each time). The aqueous solution was filtered, defatted with PE (3×500 mL), basified with 25% NH_4_OH to pH 10 and extracted three times with EtOAc (3×1 L) to afford the basic extract. Some 6.5 g of solubles were obtained. Most of the fraction consisted of saccharose, filtered off after standing overnight in MeOH:water 1:1.

### HPLC-DAD Analysis

The PEE, acid-base extraction and fractions from *G. avellana* were analyzed by HPLC coupled to a diode array detector (DAD) using a Shimadzu equipment (Shimadzu Corporation, Kyoto, Japan), as previously described^2^. An Inertsil ODS-3 RP-18 column was used for all analysis (GL Sciences Inc., Tokyo, Japan; 250 mm ×4.6 mm, 5 µm). Separation was carried out using MeOH (solvent A) and H_2_O:formic acid (solvent B, 99.9:0.1 v/v). The chromatographic conditions were: 5% A: 95% B from 0 to 2 min, 20% A: 80% B at 8 min; 52% A: 48% B at 40 min; 75% A: 25% B at 45 min; 100% A from 50 to 55 min and return to 5% A and 95% B at 60 min; flow rate: 0.8 mL/min, T° 30 °C. The chromatograms were monitored at 254, 280 and 330 nm and the UV/visible spectra were recorded from 200 to 600 nm.

### Counter-current Chromatography

A J-type Quattro MK5 Lab Prep (AECS, Wales, UK) counter-current chromatography (CCC) equipment was used. The equipment characteristics were described in Mieres-Castro *et al*.^6^. The biphasic solvent systems evaluated consisted of TBME/1-BuOH/ACN/H_2_O in different proportions, acidified with 0.1% of trifluoroacetic acid. The partition coefficients (K_*D*_) were determined as follows: 5 mg of the sample was dissolved in 4 mL of each one of the pre-equilibrated biphasic solvent system assayed (1:1 v/v). The mixture was dissolved, agitated in vortex and left to equilibrate for 2 min. When two clear phases were observed, 1 mL of each phase was taken, evaporated under reduced pressure, suspended in 0.5 mL of the mobile phase of the HPLC solvents and injected into the HPLC-DAD system described above. The K_*D*_ was calculated as the ratio of peak areas of target compounds in the upper phase, divided into the peak areas found in the lower phase (*head-to-tail* mode)^7^, by dividing the area of the main compounds found in the upper phase by the area found in the inferior phase in the HPLC-DAD system described above. All separations were carried using the organic upper phase as the stationary phase. The CCC separation was repeated two times with 657 and 573 mg of the PEE, respectively. Temperature was set at 30 °C, flow rate of 4 mL/min and revolution speed of 650 rpm. Fractions were collected with a Gilson FC 203B (Middleton, WI, USA) set at 1.0 min/tube. To end the CCC, rotation was stopped and the column content was pushed out of the system using 600 mL of a mixture MeOH:H_2_O (6:4, v/v). The components of each fraction were visualized by means of thin-layer chromatography (TLC) using Alugram^®^ plates (Macherey-Nagel GmbH & Co, Düren, Germany). The mobile phase consisted of EtOAc:formic acid:H_2_O (7:1.5:1, v/v/v), and the plate was revealed with *p-*anisaldehyde-sulfuric acid. Fractions with similar TLC patterns were combined and taken to dryness under reduced pressure. The fraction was freeze-dried, and the resulting solid was used for spectroscopic and spectrometric characterization.

### NMR, MS and IR Analysis

The NMR spectra were recorded on a Bruker Avance 400 spectrometer (Bruker, Rheinstetten, Germany) at 400 MHz for ^1^H and 100 MHz for ^13^C in CD_3_OD. Chemical shifts are given in ppm with residual methanol as the internal standard. The HR-ESI-MS-QTOF analyses were carried out using a Micromass Q-TOF instrument (Manchester, UK). The samples were infused directly using a syringe pump (Harvard Apparatus, Holliston, MA, USA) at a flow rate of 10 µL/min. The mass spectra were measured in the positive ion mode. The infrared (IR) spectra were measured using a Nexus Nicolet 470 Fourier-Transform IR (FT-IR) transmission spectrophotometer (Thermo Nicolet Corp, Madison, WI, USA).

## Results and Discussion

**Figure 2:**
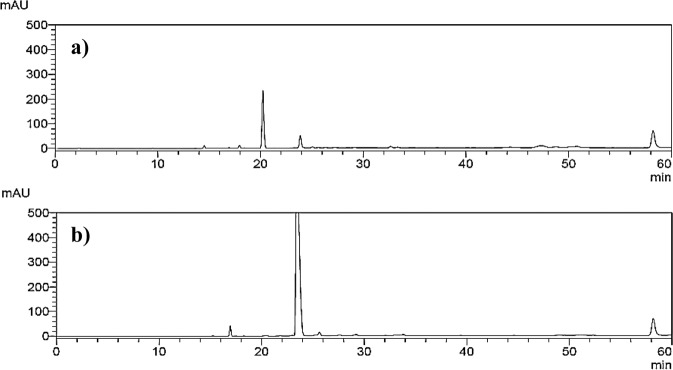
HPLC-DAD chromatogram of the fractions c and e obtained by counter-current chromatography of the *Gevuina avellana* hazelnut extract. (**a**) Fraction 81–95 (compound **c**); (**b**) Fraction 186–200 (compound **e**).

Counter-current chromatography (CCC) was used to isolate compounds present in the PEE from *G. avellana* cotyledons. CCC is a method based on the partition of analytes between two immiscible liquid phases to obtain a suitable separation, avoiding a solid support and irreversible adsorption^7^. The K_*D*_ values of main compounds from *G. avellana* cotyledons are shown in Table 1. The system composed of 2.2:2.2:0.1:5.5 (TBME:1-BuOH:ACN:H_2_O, v/v/v/v) was selected, showing K_*D*_ values suitable for CCC separation. After setting the CCC under the selected experimental conditions, 80% retention of the stationary phase was achieved. The fractions with similar TLC patterns were combined and taken to dryness under reduced pressure. The w/w extraction yields of compounds/fractions **c** and **e** from the starting PEE was 1.86 and 4.64%, respectively. Compound **c** eluted in tubes 81–95 (320–380 mL, 23 mg) (Fig. 2a), while compound **e** was found in tubes 186–200 (744–800 ml, 57 mg) (Fig. 2b). Other fractions and the extrusion presented mixtures of phenolic compounds and phenylpropanoids previously reported in Pino Ramos *et al*.^2^.

**Table 1:** Summary of the K_*D*_ values of biphasic solvent system (TBME/1-BuOH/ACN/H_2_O) for the main peaks of *G. avellana* PEE. Compounds: a: caffeic acid hexoside; b: hydroxymethylfurfural; c: mixture; d: phenyl caffeate; e: cyclo(Arg-Trp); f: hydroxybenzoic acid; g: sinapic acid hexoside; h: benzoic acid.

TBME:n-BuOH: ACN:H_2_O +0.1% TFA	K_D_ Peak a (Rt. 14.5 min)	K_D_ Peak b (Rt. 16.9 min)	K_D_ Peak c (Rt. 20.1 min)	K_D_ Peak d (Rt. 22.8 min)	K_D_ Peak e (Rt. 23.7 min)	K_D_ Peak h(Rt. 34.8 min)
3:1:1:5	0.24	0.21	0.30	0.89	0.64	2.77
2.75:1.25:1:5	0.43	0.36	0.59	1.46	1.37	4.88
2.2:2.2:0.1:5.5	0.33	0.11	0.79	2.18	2.48	10.07
1.8:1.8:2.1:4.3	1.16	1.13	1.86	2.54	2.99	5.11

The ^1^H-NMR spectrum of fraction **c** showed typical signals for tryptophan as the main constituent. The QTOF-MS analysis of this fraction showed the exact mass of 205.0978, in agreement with the molecular formula C_11_H_13_N_2_O_2_^+^, calculated as 205.0972 (error: 2.9 ppm) and fragment ions at *m/z* 143.0707 (29), 118.0659 (100), 115.0536 (78), 91.0535 (18), 146.0599 (10), 143.0742 (24), 118.0652 (100), 115.0544 (75), 91.0542 (40), confirming the occurrence of tryptophan.

The IR spectrum of the compound **e** showed absorption bands at 3352 (NH of amide), 2926, 2857, 1667 (carbonyl, cyclic amide), 1534, 1433, 1199 and 1138 cm^−1^, suggesting the presence of alkaloids or amino acids/peptides. The ^1^H-NMR spectrum of **e** (Table 2) showed characteristic signals of aromatic H from a heterocycle at *δ* 7.55 d (7.6), 7.32 d (7.6), 7.08 t (7.6, 7.2), 7.00 t (7.6, 7.2) and 7.09 (s) ppm, a pair of dd at *δ* 3.15 and δ 3.37 ppm and a H at *δ* 4.75 dd, suggesting a tryptophan derivative. Additional signals at *δ* 3.36 (m) and three CH_2_ m at *δ* 3.00, 2.21 and 1.79 ppm, indicated a second amino acid unit. The ^13^C-NMR spectrum showed 17 signals, including two carbonyls at *δ* 173.99 and 173.77 ppm, eight aromatic sp^2^ carbons from the tryptophan and two C doublets at *δ* 48.17 and 53.26 ppm, suggesting a diketopiperazine formed from tryptophan and a second amino acid. The sp^2^ C at 157.26 ppm supports the presence of the amino acid arginine. Correlation spectra allowed the identification of the compound as the dipeptide cyclo(Arg-Trp), in agreement with the proposed structure and with literature data. The NMR data and structure of the compound are summarized in Table 2. The cyclic dipeptide cyclo(Arg-Trp) was previously described by NMR and chiral spectroscopy studies on the absolute configuration and conformation of cyclic dipeptides^8^.

**Table 2:** ^1^H and ^13^C NMR data of cyclo(Arg-Trp) from *Gevuina avellana* cotyledons. Pos. Position; COSY: correlation spectroscopy; NOESY: Nuclear Overhauser Effect Spectroscopy; HSQC: heteronuclear single-quantum correlation spectroscopy; HMBC: heteronuclear multiple-bond correlation spectroscopy.


Pos.	δ_H_ (*J* in Hz)	COSY	NOESY	^13^C/HSQC	HMBC
1	3.36 *m*			48.17 d	
3	—			173.99 s	
4	4.75 *dd* (8.4, 4.4)	H12	H12	53.26 d	C12, C13
6	—			173.77 s	
7	2.21 *m* (2 H)	H8	H8, H9	31.66 t	C6, C8, C9
8	1.70 *m* (2 H)	H7, H9	H7, H9	24.37 t	C6, C7, C9
9	3.00 *m* (2 H)	H8	H7, H8	40.11 t	C7, C8, C11
11	—			157.26 s	
12	3.37 *dd* (14.6, 4.4)3.15 *dd* (14.6, 8.8)	H4, H12, H14	H4	27.06 t	C3, C4, C13, C14, C17
13	—			109.67 s	
14	7.09 *s*	H12		123.06 d	C12, C16, C17
16	—			136.62 s	
17	—			127.38 s	
18	7.55 *d* (7.6)	H19	H19	117.81 d	C13, C16, C17, C20
19	7.00 *dd* (7.6, 7.2)	H18	H18, H20	118.43 d	C17, C21
20	7.08 *dd* (7.6, 7.2)	H21	H19, H21	121.04 d	C16, C18
21	7.32 *d* (7.6)	H20	H20	110.94 d	C17, C19

Cyclic dipeptides are 2,5-diketopiperazines that are found as natural products in a several sources, including bacteria, yeast, fungi, plants and mammals^9^. They can also be formed during chemical and thermal processing of food products^10^, including the roasting process^11,12^. Cyclic dipeptides contribute to the final taste of food, increasing astringency, saltines, bitterness and metallic flavors^9^. In addition, they have shown several bioactivities, including antibacterial, antifungal and antitumoral effects^13^. The recent review of Borthwick and Da Costa^9^ list the occurrence of cyclic dipeptides in food and beverages. To the best of our knowledge, this is the first time that this compound is reported in *Gevuina avellana* cotyledons. The Cyclo(Arg-Trp) was isolated from roasted cotyledons of *G. avellana*; however, its presence was confirmed also in the acid-base extraction of the cotyledons.

In summary, tryptophan and a cyclic dipeptide were identified from *G. avellana* cotyledons. Arginine, aspartic acid, leucine and tryptophan were the main amino acids in the hydrolysate of Chilean hazelnut^5^. Tryptophan is an essential amino acid occurring in most of the peptides in *G. avellana* cotyledons and plays a relevant role on cognition and mood, with positive effects in memory and attention^14^. In addition, Trp is a key amino acid in the biosynthesis of serotonin and melatonin, as well as kynurenin and their derivatives^15^. The relevance of tryptophan-rich foods on nutrition and psychiatric symptoms has been recognized^16^. The relation of Trp with depression and obesity^17^, food craving^18^, food intake and microbiota^19^ has been also studied. The content of serotonin in several nuts, including Macadamia nuts, was recently determined but no information is available on the serotonin content in Chilean hazelnuts^20^. Additional studies are needed to fully disclose the possible beneficial effects of the *G. avellana* nuts in human health.
